# Sexually Transmitted *Trichophyton mentagrophytes* Genotype VII Infection among Men Who Have Sex with Men

**DOI:** 10.3201/eid2907.230025

**Published:** 2023-07

**Authors:** Arnaud Jabet, Sarah Dellière, Sophie Seang, Aziza Chermak, Luminita Schneider, Thibault Chiarabini, Alexandre Teboul, Geoffroy Hickman, Alizée Bozonnat, Cécile Brin, Marion Favier, Yanis Tamzali, François Chasset, Stéphane Barete, Samia Hamane, Mazzouz Benderdouche, Alicia Moreno-Sabater, Eric Dannaoui, Christophe Hennequin, Arnaud Fekkar, Renaud Piarroux, Anne-Cécile Normand, Gentiane Monsel

**Affiliations:** Assistance Publique-Hôpitaux de Paris, Paris, France (A. Jabet, S. Dellière, S. Seang, A. Chermak, L. Schneider, T. Chiarabini, A. Teboul, G. Hickman, A. Bozonnat, C. Brin, M. Favier, Y. Tamzali, F. Chasset, S. Barete, S. Hamane, M. Benderdouche, A. Moreno-Sabater, E. Dannaoui, C. Hennequin, A. Fekkar, R. Piarroux, A.-C. Normand, G. Monsel);; Université de Paris, Paris, France (S. Dellière, E. Dannaoui);; Sorbonne Université, Paris, France (F. Chasset, A. Moreno-Sabater, C, Hennequin, A. Fekkar, R. Piarroux)

**Keywords:** Tinea, sexually transmitted infections, Trichophyton mentagrophytes, monkeypox, HIV, terbinafine, dermatophytosis, fungi, viruses, France

## Abstract

Transmission of dermatophytes, especially *Trichophyton mentagrophytes* genotype VII, during sexual intercourse has been recently reported. We report 13 such cases in France. All patients were male; 12 were men who have sex with men. Our findings suggest sexual transmission of this pathogen within a specific population, men who have sex with men.

Dermatophytes are keratinophilic fungi responsible for frequent skin infections. They are transmitted either by direct contact from an infected host (human or animal) to a receptive host or from the environment. In 2002, two surveys reported cases of tinea cruris infection in sex workers, raising the hypothesis of sexual transmission of dermatophytes (*1*,*2*). In 2009, transmission of *Trichophyton mentagrophytes*, responsible for tinea genitalis, between a heterosexual couple was reported (*3*). Subsequently, a specific internal transcribed spacer (ITS) genotype of *T. mentagrophytes*, genotype VII (TMVII), was reported for cases of suspected sexual transmission, most frequently tinea genitalis (*4*–*7*). In some cases, a temporal association was demonstrated between the appearance of lesions and sexual intercourse between occasional partners, especially sex workers in Southeast Asia (*4*,*8*). Moreover, similar lesions were repeatedly documented in sex partners of infected patients (*4*,*6*). Unlike other *T. mentagrophytes* genotypes, TMVII has not been reported in association with dermatophytosis in children in contact with animals (*9*). We report 13 cases of TMVII infections, highly suspected of being sexually transmitted, diagnosed in 3 large tertiary care hospitals in Paris, France, in men who have sex with men.

## The Study

During January 2021–September 2022, for all strains that could correspond to *T. mentagrophytes* or *T. indotineae* that were isolated at La Pitié-Salpêtrière and Saint-Antoine Hospitals in Paris, we sequenced the ITS1–5.8S-ITS2 region for species identification and genotype determination (*10*). At Saint-Louis Hospital in Paris, sequencing was limited to the isolates responsible for widespread dermatophytosis. For all cases of confirmed TMVII infection, we retrieved the medical records.

Of the 13 cases of TMVII infection, the first was detected in March 2021, and 9 were diagnosed during June–September 2022. All patients were male; median age was 39 (22–59) years (Table 1). Five patients had a single skin lesion, and others had multiple lesions. One patient had inguinal papules and nodules suggestive of Majocchi granulomas, 2 had highly inflammatory folliculitis of the beard (kerion), and the others had typical erythemato-squamous lesions with an active border (Figure).

**Table 1:** Main epidemiologic and clinical features of 13 cases of *Trichophyton mentagrophytes* genotype VII infections diagnosed in Paris, France, 2021–2022*

Pt no.	Age, y	HIV+	PrEP	STI history	Travel	Tinea genitalis	Tinea glutealis	Tinea corporis	Tinea faciei/barbae	Prior treatment	*T. mentagrophytes* treatment
1†	45	No	Yes	Ng, Ct, Mg	No	No	Yes	Yes	Yes	No	TRB 1 mo
2	34	No	Yes	Ng	EE	No	Yes	Yes	Yes	ECZ, TS	TRB 5 d, then ITR 200 mg 1 mo, then ITR 100 mg 1 mo
3	28	No	No	ND	ND	Yes	No	Yes	No	No	TRB 4 mo + BFZ 1 mo
4	59	Yes	NA	Ng, Ct, Mg, Tp, HCV	No	Yes	Yes	Yes	Yes	No	TRB 2 mo + ECZ
5‡	39	Yes	NA	Tp	ND	Yes	Yes	Yes	Yes	No	TRB + CPX 3 wk
6‡	41	Yes	NA	Tp	ND	No	Yes	Yes	Yes	No	TRB + CPX 3 wk
7	40	No	Yes	Ng, Ct, Tp	No	No	No	No	Yes	PRI + MPC	TRB 6 wk
8	48	Yes	NA	Ng, Ct, Tp, Ss	No	No	No	Yes	No	No	CPX 4 wk
9‡	26	Yes	NA	Ng, Ct, Tp	ND	Yes	Yes	Yes	No	No	ECZ 6 wk
10‡§	35	No	Yes	Tp	ND	No	No	Yes	No	No	ECZ 6 wk
11§	22	No	Yes	Ng, Ct, Tp	DE	No	Yes	Yes	Yes	AMX then FLC	TRB 4 wk
12	35	Yes	NA	Ng,Tp	IN	No	No	Yes	No	TS then CPX	BFZ 4 wk
13	46	Yes	NA	Ng, Ct, Tp, Ss	ES	No	No	No	Yes	FCD + TS then FCD alone then PRI then AMX/CLAV	ITR 100 mg 2 d, then IV VRC 10 d, then TRB

*AMX, amoxicillin; BFZ, bifonazole; CLAV, clavulanic acid; CPX, ciclopirox olamine; Ct, *Chlamydia trachomatis*; DE, German; ECZ, econazole; EE, Estonia; ES, Spain; FCD, fucidin; FLC, fluconazole; HCV, hepatitis C virus; IN, India; ITR, itraconazole; Mg, *Mycoplama genitalium*; MPC, mupirocin; NA, not applicable; ND, no data; Ng; *Neisseria gonorrheae*; PrEP, pre-exposure prophylaxis; PRI, pristinamycin; Pt, patient; Ss, *Sarcoptes scabiei;* Tp, *Treponema pallidum*; TRB, oral terbinafine (250 mg 1×/d); TS, topical steroids; VRC, voriconazole. †Patient 1 strain was included in a previous survey (*11*). ‡Patients 5 and 6 and patients 9 and 10 were partners. §Patients 10 and 11 were co-infected with monkeypox virus.

**Figure:**
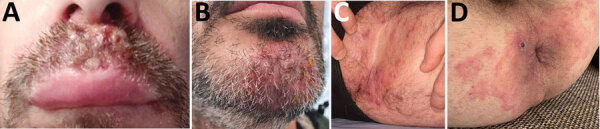
Clinical appearance of *Trichohpyton mentagrophytes* genotype VII infections in men in France, 2022. A, B) Swollen lesions of the mustache (A) and beard (kerions) (B). C) Papular and nodular inguinal lesions. D) Peri-anal mpox lesions with associated papules and pustules with central umbilication and a large lesion with a central necrotic crust, surrounded by extensive erythemato-squamous circinate lesions caused by TMVII infection.

Of the 13 patients, 11 reported having sexual relations exclusively with men and 1 reported having sexual intercourse with men and women. At least 9 had multiple sex partners in the month before lesion onset. Seven patients were HIV positive, and 5 were taking HIV preexposure prophylaxis. Apart from 1 patient who had recently discontinued treatment, all HIV-positive patients were receiving long-term effective antiretroviral treatment. Twelve patients had previously experienced sexually transmitted infections (STIs) other than HIV. STI testing was performed for 8 patients, leading to detection of *Chlamydia trachomatis* DNA in an anal sample for 1 patient and a syphilis diagnosis for 1 patient. Two patients were co-infected with monkeypox virus, and mpox developed in another patient 1 month after the dermatophytosis diagnosis. For 1 patient, TMVII infection and mpox lesions appeared concurrently in the peri-anal region, suggesting transmission of both agents at the same time (Figure).

Four patients denied any travel outside of France, 1 patient was infected in Germany (Munich) where he lived, and for 3 others, lesions developed after they had returned from travel (to Slovenia, Spain, and India). Only 3 patients reported having had contact with animals (cats or dogs). For 2 patients, both sex partners had documented TMVII infection; for 2 others, contamination from sex partners who had similar skin lesions was suspected. Likewise, 2 patients reported secondary appearance of lesions on the skin of sex partners. However, infection was not medically confirmed.

The median time between lesion appearance and hospital consultation was 28 (range 7–102) days. Five patients had previously received antibiotic, antifungal, or topical steroid treatments. Nine patients received systemic antifungal treatment (terbinafine, itraconazole, or voriconazole) for 3 weeks to 4 months; the others received only topical treatment. One patient with beard kerion and bacterial superinfection (*Klebsiella aerogenes*) was hospitalized. Among the 13 patients, 10 recovered while receiving antifungal therapy, 1 was still receiving treatment at most recent follow-up, and the 2 others were not available for further follow-up. At least 3 patients experienced postinflammatory pigmentation; 2 others had scars or beard hair loss.

During the study period, of 70 strains sequenced, the predominant identified agent was *T. indotineae* (n = 53), followed by TMVII (n = 13) (GenBank accession no. OK632215, ON740661, OP876812–22). The remaining strains corresponded to *T. mentagrophytes* genotype II*.

## Conclusions

For most patients in this series, sexually transmitted dermatophytosis was likely. This hypothesis is supported by the sites of the infection (external genitalia, buttocks, face), the high-risk STI profile of the patients, and consistent identification of TMVII. Lack of animal contact for most patients also suggests human-to-human transmission. Moreover, although *T. mentagrophytes* is considered to be a zoophilic species, no animal-to-human transmission has been documented for TMVII and only 1 strain has been isolated from an animal (cat) (*6*). All 13 patients were male, 12 of whom were men who have sex with men. Even if we cannot exclude recruitment bias, the study suggests active circulation of the pathogen within this population. Previous studies reported TMVII infections in men and women whose sexual practices were either heterosexual or not mentioned. Our series demonstrates that TMVII infected the same population of patients as did the monkeypox virus during the 2022 outbreak (*12*). Contrary to previous reports (*4*–*6*), intimate shaving was not associated with TMVII infection in our case series.

Patients might have acquired TMVII infections in France or internationally, supporting the hypothesis of active circulation of TMVII in Europe, in line with the 51 cases reported since 2014 (Table 2). Southeast Asia might have been the starting point of pathogen spread, as suggested by the first reported cases in Europe being associated with travel to that region (*4*–*6*,*8*). Of 37 cases of TMVII infection reported in Berlin, Germany, over 18 months (January 2016–July 2017), only a small proportion of documented cases was associated with travel outside Germany, suggesting that TMVII was already circulating in Europe (*6*).

**Table 2:** Cases of *Trichophyton mentagrophytes* genotype VII infections in Europe, 2014–2019*

Reference	Year	Location	Age, y/sex	No. cases	Tinea genitalis	Tinea corporis/ inguinalis	Tinea faciei/ barbae	Tinea glutealis	Comments
(*4*)	2014	Zurich, Switzerland	27/F	NA	Yes	Yes	No	No	Sexual intercourse 1–2 wk before symptom onset, Southeast Asia; similar lesions in 1 sex partner
	2014		24/M	NA	Yes	Yes	No	No	
	2014		31/M	NA	Yes	No	No	No	
(*13*)	2014	St. Petersburg, Russia	ND	NA	No	Yes	No	No	None
(*5*)	ND	Germany	ND/F	NA	Yes	Yes	No	No	Lesions appeared after return from travel in Egypt; indirect transmission was suspected
(*8*)	ND	Germany	ND/M	NA	No	No	Yes	No	Sexual intercourse with female sex workers in Thailand; secondary case in wife’s patient (tinea faciei)
(*6*)	2016– 2017	Berlin, Germany	20–40/ mostly young men	37	Most frequent presentation was tinea genitalis				4/18 persons traveled in Southeast Asia 5/18 persons had sex partner with similar lesions
(*9*)	2016	Zurich, Switzerland	21/M	NA	Yes	Yes	Yes	No	None
	2016		33/F	NA	Yes	Yes	No	No	
	2018		65/M	NA	No	No	Yes	No	
	2018		50/M	NA	No	No	Yes	No	
(*7*)	2010– 2019	Athens, Greece	ND	4	Yes	No	No	No	None

*NA, not applicable; ND, no data.

Our case series is characterized by a substantial delay in diagnosis. Patients with the most inflammatory lesions were initially mistakenly believed to have had bacterial infections (*4*,*8*). Moreover, prolonged systemic antifungal treatments and patient hospitalizations highlight how severe TMVII infections can be (*4*–*6*,*8*). Therefore, healthcare professionals should be aware of the various features of TMVII infection and perform targeted mycological samplings. In contrast to findings for *T. indotineae* (*14*), no terbinafine resistance has been reported for TMVII. Of note, during the study period, *T. indotineae*, which was recently described as an emerging species of dermatophyte in Paris, responsible for difficult-to-treat tinea (*14*), was the main agent of skin dermatophytosis caused by *T. mentagrophytes* complex species.

Transmission of dermatophytes during sexual intercourse is an example of direct human-to-human transmission, as previously described for combat sports (tinea gladiatorum) (*15*). Sexual transmission should be suspected for patients with STI risk factors and tinea corporis, tinea genitalis, tinea glutealis, or tinea faciei. Diagnosis of sexually transmitted dermatophytosis should lead to exhaustive STI screening of patients and their sex partners.
